# Opt-out cervical cancer screening increases coverage of cervical cancer screening in HIV clinical care settings: Experiences from Mildmay, Uganda

**DOI:** 10.1186/1472-6963-14-S2-O26

**Published:** 2014-07-07

**Authors:** Anne M Nabukenya, Joseph Matovu, Joan Nangiya, Esther Kawuma, Mary Odiit, Barbara Mukasa

**Affiliations:** 1 MakSPH-CDC Fellowship Program, Makerere University School of Public Health, Kampala, Uganda; 2 Mildmay Uganda, Kampala, Uganda

## Background

Cervical cancer is highly prevalent in developing countries. In Uganda, overall incidence rate is estimated at 44 per 100,000 women and 60 per 100,000 among HIV infected women. However, only 30% of women have ever been screened for cervical cancer and linkage to treatment after diagnosis is suboptimal. For HIV infected women, who are at highest risk of cervical cancer, the HIV care delivery system provides an opportunity for cervical cancer services. In this paper we share the Mildmay Uganda (MUg) experiences in integrating cervical cancer services into HIV care.

## Program implementation

The MUg clinic is a centre of excellence that has been delivering HIV clinical care since 1998 and has supported over 60,000 clients to date. In 2008, MUg started screening the HIV infected women for pre-cancerous lesions using PAP smears, and eventually introduced visual inspection with acetic acid (VIA) in 2011. Screening is conducted by trained nurses through an opt-out model and an onsite see-and-treat (same-day treatment) with cryotherapy for women with growing lesions.

## Program outcomes

The introduction of the opt-out model and screening by nurses has led to drastic increases in the number of women screened and treated for cervical cancer since 2012. In 2011, a total of 730 women were screened, while in 2012, 3,857 women were screened. In 2013, 2,580 women were screened. Overall, refusal of screening was less than 5%. The number of women observed with advanced cancerous lesions has drastically reduced since introduction of the opt-out screening. For example, in the first half of 2012, of the 96 women who were VIA positive, 56% had advanced lesions compared to 41% in the latter half of 2012, 32% in the first half of 2013, and 15% in the latter half of 2013.

## Lessons learnt

Integration of cervical cancer services in HIV care programs is possible, cost-effective and makes the screening service more accessible by a vulnerable population. Using an opt-out approach and same-day treatment increases access to cervical cancer screening and reduces the proportion of women presenting with advanced lesions. Trained nurses ably deliver cervical cancer services. See-and-treat approach reduces cases who fail to access treatment due to gaps in health care linkages.

